# A system for probing Casimir energy corrections to the condensation energy

**DOI:** 10.1038/s41378-020-00221-2

**Published:** 2020-12-28

**Authors:** Diego Pérez-Morelo, Alexander Stange, Richard W. Lally, Lawrence K. Barrett, Matthias Imboden, Abhishek Som, David K. Campbell, Vladimir A. Aksyuk, David J. Bishop

**Affiliations:** 1 Department of ECE, Boston University, Boston, MA 02215 USA; 2 Physical Measurement Laboratory, National Institute of Standards and Technology, Gaithersburg, MD 20899 USA; 3 Institute for Research in Electronics and Applied Physics & Maryland NanoCenter, University of Maryland, College Park, MD 20742 USA; 4 Division of MSE, Boston University, Boston, MA 02215 USA; 5 4K-MEMS Sàrl, Rue des Lavannes 17, 2072, St Blaise, Switzerland; 6 Department of Physics, Boston University, Boston, MA 02215 USA; 7 Department of ME, Boston University, Boston, MA 02215 USA; 8 Department of BME, Boston University, Boston, MA 02215 USA

**Keywords:** Physics, Materials science, Nanoscale materials, NEMS

## Abstract

In this article, we present a nanoelectromechanical system (NEMS) designed to detect changes in the Casimir energy. The Casimir effect is a result of the appearance of quantum fluctuations in an electromagnetic vacuum. Previous experiments have used nano- or microscale parallel plate capacitors to detect the Casimir force by measuring the small attractive force these fluctuations exert between the two surfaces. In this new set of experiments, we aim to directly detect the shifts in the Casimir energy in a vacuum due to the presence of the metallic parallel plates, one of which is a superconductor. A change in the Casimir energy of this configuration is predicted to shift the superconducting transition temperature (*T*_c_) because of the interaction between it and the superconducting condensation energy. In our experiment, we take a superconducting film, carefully measure its transition temperature, bring a conducting plate close to the film, create a Casimir cavity, and then measure the transition temperature again. The expected shifts are smaller than the normal shifts one sees in cycling superconducting films to cryogenic temperatures, so using a NEMS resonator in situ is the only practical way to obtain accurate, reproducible data. Using a thin Pb film and opposing Au surface, we observe no shift in *T*_c_ >12 µK down to a minimum spacing of ~70 nm at zero applied magnetic field.

## Measuring quantum fluctuations with NEMS

A nano-electromechanical system (NEMS) allows for the direct measurement of Casimir energy. The Casimir effect relates to the fluctuation of electromagnetic waves between two plates in a vacuum. Previous studies used plate capacitors that function by detecting small Casimir forces exerted on the plates by the electromagnetic fluctuations. Now, a team from the United States and Switzerland led by David Bishop, of Boston University, Massachusetts, and Vladimir Aksyuk of the National institute of Standards and Technology, have developed a technique in which a thin lead superconductor film is placed parallel to a gold surface. This allows direct measurements as the plate and film are brought close together and the actual Casimir energy in the cavity changes. The team measured the Casimir energy via its effect on the superconductor’s “transition temperature”, finding changes no more than 12 micro-Kelvin. The team says their paper “opens a novel experimental window” that future studies should exploit.

## Introduction

The Casimir force was first derived in 1948 by calculating the van der Waals force using retarded potentials^1^. This force is a purely quantum mechanical force that arises between two plates even when they are not electrically charged. Classically, there is no force on the plates. However, due to quantum fluctuations and the freezing out of the long-wavelength electromagnetic modes, there is a net pressure exerting an attractive force. Experimentally, the effect has been seen using a number of microscale systems and devices^2–8^. Reference^4^ discusses how the force varies with the metallic conductivity of the plates. References^9,10^ show how the effect can be used for practical applications and refs. ^11,12^ show how a repulsive force can be achieved. Additional work has also demonstrated the importance of nanopatterning^13^ and magnetic effects^14^.

Given that the metallic conductivity changes the magnitude of the Casimir force, the question immediately comes to mind “what happens if the plates become superconducting?” The answer disappointingly is “not much.” The Casimir effect averages the conductivity of the material over very large energy scales, while the superconducting gap is relevant only for the far infrared^15^. Therefore, while the effect of superconductivity is very large (100%) on the DC conductivity, it is negligible and unmeasurable if averaged over the typical energy scales found in a Casimir cavity^16^. Therefore, one cannot see an effect on the measured Casimir force at the transition temperature *T*_c_.

However, as pointed out in work by Bimonte and coworkers^17^, one might be able to see an effect of Casimir energy on the superconductivity. In a type I superconductor, the critical parallel field *H*_c||_(*T*) is given by the change in the free energy, Δ*F*, which is the difference between the free energy in the superconducting and normal states:

1 $$\left( {{{H}}_{{\mathrm{c||}}}\left( {{T}} \right)} \right)^2 \propto \Delta {{F}}\left( {{T}} \right)$$

Reference^17^ suggests that this free energy change may also be related to the Casimir energy:

2 $$\Delta {{F}} = {{E}}_{{\mathrm{cond}}}\left( {{T}} \right) + \Delta {{E}}_{{\mathrm{Cas}}}\left( {{T}} \right)$$

where *E*_cond_(*T*) is due to the superconductivity and Δ*E*_Cas_(*T*) is due to the Casimir energy. For small modulations in this Casimir term, the critical field is modulated by a factor proportional to the ratio Δ*E*_Cas_/*E*_cond_. The calculations performed in ref. ^18^ suggest that this fraction could be as large as 10% in certain thin-film materials with low condensation energies.

**Fig. 1:**
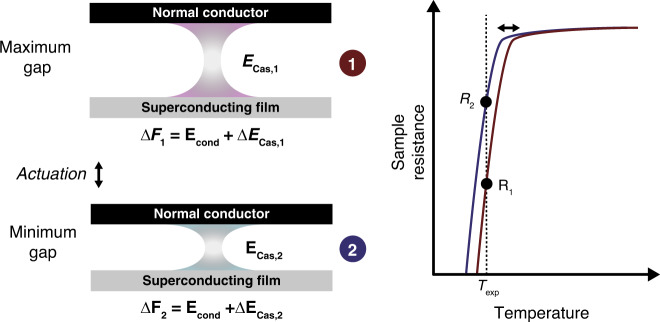
Basic concept of our experiment. Holding the system at temperature *T*_exp_, just below the transition, a conducting plate is actuated in close proximity to a superconductor to modulate the Casimir contribution to its total free energy change of condensation (Δ*F*), and thereby *T*_c_. The two positions shown correspond to the maximum and minimum gap sizes due to an oscillation of the plate

The theory suggests the following: first, placing a superconducting film in a Casimir cavity shifts *T*_c_. Second, measuring the effect is contingent upon keeping *E*_cond_ constant. Therefore, any attempt to compare several different films, some inside cavities and some not, may suffer from uncertainty due to variations in the highly process-dependent characteristics of superconducting thin films. Even on a single die, different regions of the deposited material may display slightly different superconducting transition temperatures due to thickness variations, local roughness, or temperature gradients during deposition. In this work, we present a technique in which a nanoelectromechanical system (NEMS) structure is used to move a plate relative to a single superconducting film in situ. The basic concept is shown in Fig. 1, in which we sit on the shoulder of the superconducting transition and actuate a nearby metallic plate, thus modulating the Casimir energy while monitoring the film resistance. Due to the sharp slope of the superconducting transition, a small change in *T*_c_ (due to a change in the Casimir energy) would manifest as a measurable change in the sample resistance. The present experimental approach can be extended to include the application of a magnetic field through the sample, allowing one to measure variations in the full *H*_c_(*T*) curve for comparison with the existing theory, which currently does not provide quantitative predictions for *H* = 0^17,18^.

There are a number of experimental challenges posed by the concept shown in Fig. 1. According to calculations performed in refs. ^17,18^, the spacing of the Casimir cavity should be in the range of a few nanometers to 100 nm, and the film thickness should be on the order of 10 nm. These characteristics place serious constraints on the choice of materials, many of which tend to ball up and form islanded microstructures when thin^19^. However, evaporating onto cryogenically cooled surfaces allows for the quenched condensation of the material, forming very smooth, amorphous films^20,21^. For this reason, an in situ deposition method is used in which the superconducting film is deposited at the chip-scale, below the superconducting transition temperature. This “fab-on-a-chip” methodology is explained further in the “Methods” section as well as in refs. ^21–25^. It is with this quenched-condensed thin film (which serves as one half of a tunable Casimir cavity) that we are able to probe changes in the Casimir energy.

## Results

Experiments are performed by assembling a target chip and a source chip into a single package. Then, the system is cooled down to cryogenic temperatures, a superconducting thin film is deposited onto the target die, and a Casimir cavity is formed. Next, the film is characterized, and the size of the Casimir cavity is dynamically tuned while monitoring the film resistance. The ex situ characterization of the film and cavity is also performed using scanning electron microscopy (SEM) and atomic force microscopy (AFM) after the experiment is complete. The following results are presented according to this sequence.

### Chip-scale evaporation and measurement setup

The non-rigid Casimir cavity imposes serious nanofabrication challenges that must be overcome to successfully obtain a functional device. First, due to process incompatibilities, we need to be able to deposit the superconducting sample underneath the metallic plate after releasing the movable structures. Second, due to the oxidation of the thin film, evaporation and resistance measurements need to be performed without breaking vacuum conditions. Finally, low temperatures can be used to reduce the migration of the evaporated material along the substrate, enabling high-quality films.

**Fig. 2:**
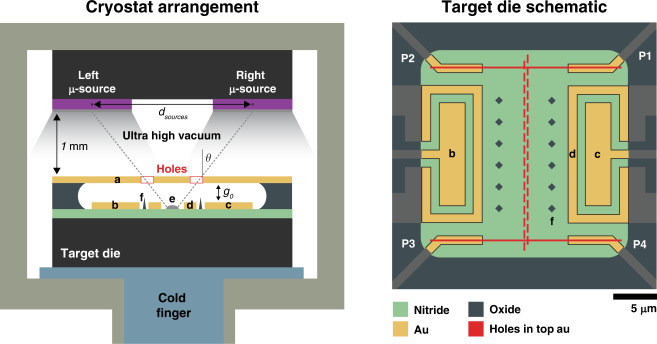
Schematic of the experimental setup and target die. Two MEMS-based microsources (purple) generate a flux of Pb atoms that is deposited onto a specially designed target die. Using angled evaporation through a suspended, patterned Au layer (**a**), a continuous film (**e**) is formed that connects the four prepositioned measurement leads (P1, P2, P3, P4). Au electrodes (**b**, **c**) are then used to actuate and sense the top suspended Au layer. Grounded guards (**d**) surround each electrode to minimize the current leakage and stray electric fields. As shown in the target schematic, the pattern etched into the top Au layer (red) acts as both the mask for evaporation and allows the suspended portion of the top Au layer to move, resulting in a tunable Casimir cavity. Silicon oxide pillars (**f**) serve as a physical stop for the movable Au plate

Figure 2 shows a schematic of the three-die arrangement developed for this experiment, consisting of one NEMS target die (onto which the material will be evaporated) centered between two microsource dies. The flux of the material being evaporated diagonally from each microsource die will reach the target die and form a continuous, thin film underneath the top Au layer. This top Au layer on the target die serves as both a physical mask and a movable plate (to vary the Casimir gap size, which is nominally *g*_0_). The bottom Au layer of the target die consists of two sets of electrical leads. The rectangular structures shown in Fig. 2 (target schematic) are the plate drive and sense electrodes for the movable gold plate, with a ground shield around them. The four leads heading off at 45° (P1, P2, P3, P4) in the target die schematic are the four-point contacts to the superconducting film used to measure its resistance. The film is formed by two angular evaporations through the holes (red) that combine to form a continuous strip along the center. Another important feature of the target die is the presence of silicon oxide pillars that serve as physical stops for the movable Au plate, both protecting the sample from contact with the Au as well as providing information regarding the minimum cavity size achieved. The microsource dies, shown in purple, are separated by distance *d*_sources_ and consist of an array of microscale heaters preloaded with a layer of superconducting material. The design and fabrication of the microsources and targets are explained further in the Supplementary Information.

### Quenched-condensed Pb thin film

After the system is cooled to ≈3 K, the microsources are slowly heated until the evaporation of Pb occurs. By applying short voltage pulses through microsources, very small amounts of material can be evaporated in a controlled manner. Because the target die is cooled to cryogenic temperatures, the Pb reaching the target forms a quenched-condensed film of just 20 nm to 30 nm (see ex situ AFM results). According to the mask pattern displayed in Fig. 2, the dual-angle deposited Pb film connecting four Au measurements leads to an “H” pattern, allowing for a four-point resistance measurement to be made.

**Fig. 3:**
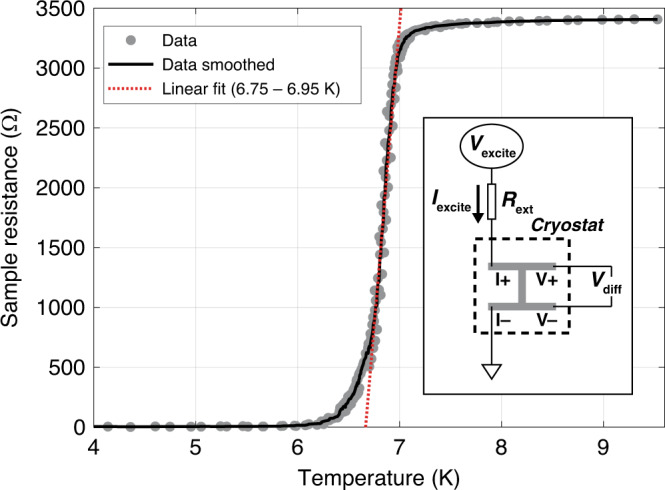
Superconducting transition of the quenched-condensed Pb film. Data points (gray dots) are taken both sweeping up and down through the transition, and the black line is the smoothed average of these data points. The slope of the transition is calculated to be (11.4 ± 0.4) kΩ/K. Inset: four-point resistance detection scheme. The current is applied through an external resistance, and the voltage drop across the Pb sample is measured

The measured resistance of the quenched-condensed Pb film as the temperature is swept from 4 to 9.5 K and back down is shown in Fig. 3. The temperature at which the film begins to condense into the superconducting phase is ≈7.05 K, just under the bulk value of 7.193 K^26^, indicating a thicker film (~20 nm^21^). The resistance of the film above *T*_c_ is ≈3.4 kΩ, and the slope at the center of the transition is (11.4 ± 0.4) kΩ/K. The uncertainty on the slope is reported from the 95% confidence bounds of the linear fitting in the range of 6.75–6.95 K.

### Cavity modulation and *T*_c_ shift measurement

**Fig. 4:**
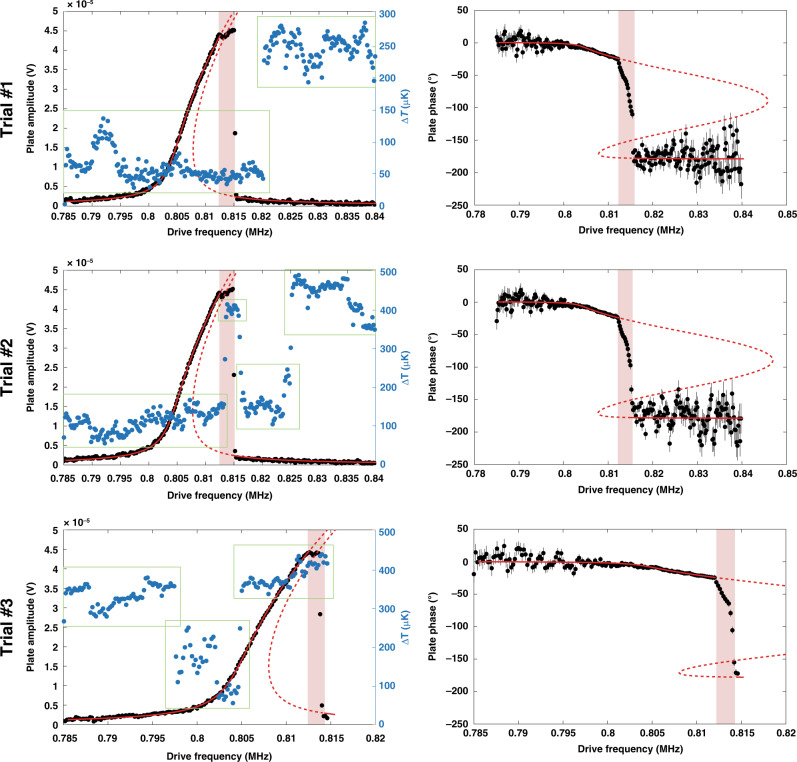
High-frequency detection of superconducting Pb film and cavity size. Black data are amplitude (left plots) and phase (right plots) of the movable plate for each trial, along with fits to a Duffing model with *ω*_0_ = 805.4 kHz and *Q* ≈ 3500 (red lines). Uncertainties for the amplitude measurements are smaller than the symbol size. Phase uncertainties are one standard deviation uncertainties propagated from the measured statistical uncertainties in the *x* and *y* quadratures. Regions, where the plate comes into contact with the oxide pillars, are highlighted in pink. As the plate is swept through its resonance, the Pb resistance is recorded and scaled to units of the temperature change (blue data) using the slope of the superconducting transition. Note that Δ*T* = 0 µK is arbitrary from plot to plot. The green boxes indicate the regions of data that are used to quantify the one standard deviation uncertainty of the Δ*T* measurements (28 µK for each point)

The experiment is carried out by setting the temperature of the cryostat to the steepest point of the transition (*T* ≈ 6.88 K), where the measured resistance is most sensitive to changes in temperature. Then, the plate drive voltage is swept through its mechanical resonance while measuring the modulation of the resistance of the sample at the mechanical motion frequency. By operating the NEMS around resonance, we cannot only produce large changes in the gap size but also perform the measurement at a high frequency (>1 MHz), which greatly reduces measurement noise. Plotted in Fig. 4 are three trials using this detection method, trial #1, trial #2, and trial #3. The black data points show the frequency dependence of the amplitude and phase of the Au plate (left and right figures, respectively). A common Duffing-nonlinear oscillator response is evident for amplitudes below the plate’s mechanical contact with the oxide pillars. The blue data points show the change in resistance of the sample expressed in units of change in the transition temperature, using the slope calculated in Fig. 3.

The abscissa of the plots in Fig. 4 is the frequency of the signal being applied at the drive electrode. Because this is a purely AC signal, the electrostatic force applied to the plate and its resulting motion is actually at twice this frequency (see “Methods” section for details on this detection scheme). This 2× frequency difference between the electrical drive and the expected superconductor modulation signal largely eliminates any direct electrical crosstalk. For each trial, the frequency is swept upwards, and the plate amplitude/phase and sample resistance are all recorded simultaneously.

**Fig. 5:**
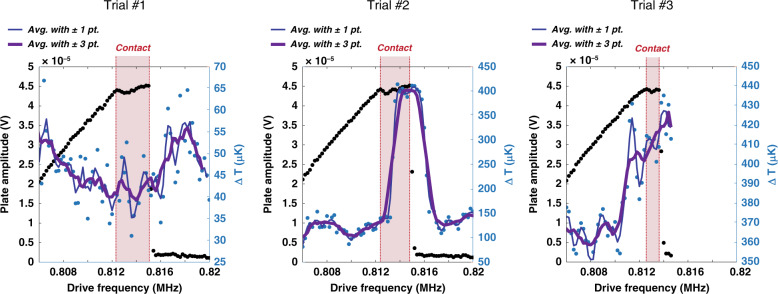
A closer look at high-frequency results. Only the regions before, during, and after contact are plotted. In addition to the data points, the sliding averages are plotted for better visualization. The one standard deviation uncertainty on Δ*T* measurements is 28 µK for each point

Additionally, the same three trials shown in Fig. 4 are plotted in Fig. 5 but are zoomed in to the region of interest (close to contact with the oxide pillars and just after the amplitude drop). In addition to plotting the data points, we include two sliding average curves to help visualize trends in the noisy data. These sliding averages take the mean of either one value or three values on either side of each data point (thin blue line and thick purple line, respectively).

### Ex situ characterization

**Fig. 6:**
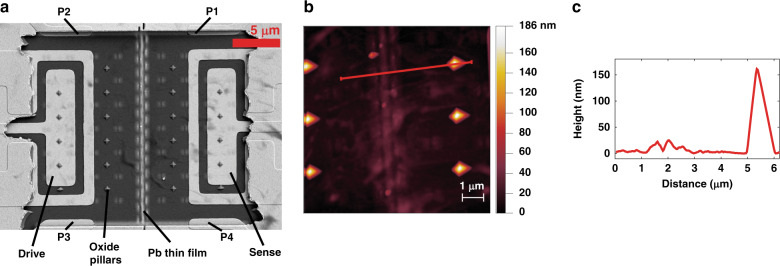
Ex situ analysis of the cavity and film. **a** SEM image of the cavity with the top Au layer removed using adhesive tape. A continuous Pb film can seem to connect the four Au leads. **b** AFM measurement of the Pb sample and several oxide stops. The height data corresponding to the red profile are plotted in **c**

After running the experiment in the cryostat, the SEM and AFM measurements of the nanocavity (with the top Au surface removed) are performed. In Fig. 6a, the features of the bottom Au layer depicted in Fig. 2 are shown, consisting of two rectangular electrodes and four measurement leads. Additionally, the deposited Pb sample is visible, connecting all four leads and forming a continuous strip down the center. Figure 6b, c shows the AFM height measurements of the sample and the oxide stops. The sample height is measured to vary between 20 and 30 nm. Using the SEM image, the length of the central portion of the sample is (20.1 ± 0.1) µm, and its width is (403 ± 57) nm. These values are obtained by averaging several measurements along the sample width and length, and the uncertainty is one standard deviation. The height of the oxide pillars is measured to be 160 ± 1 nm from averaging the heights of the six measured pillars shown in Fig. 6b, and the uncertainty is one standard deviation.

Because the plate comes into contact with the oxide pillars, it is possible to estimate the vertical displacement of the center of the plate; however, to accomplish this, an assumption of the shape of the deformed Au must be made. The difficulty of this estimate is that the stress state of the suspended Au layer at cryogenic temperature is unknown. Using a series of finite element simulations, a likely range of the minimum Au/Pb spacing (i.e., when the amplitude of the plate is maximum) is estimated to be between 63 and 73 nm. Across the entire length of the sample, the average Au/Pb spacing at maximum amplitude is estimated to lie between 85 and 141 nm. This analysis is explained further in the “Methods” section.

## Discussion

We have developed a platform based on a variable gap cavity including an Au nanoscale mechanical resonator and a high-quality quenched-condensed superconductor film, enabling the simultaneous electromechanical control of the nanometric gap between plates and the transport measurement of the superconducting film using a four-probe configuration. The high-frequency detection method presented makes use of the natural resonance of the plate to obtain the large modulations of the cavity size and allows for the phase-sensitive detection of changes in the sample resistance due to the plate motion only.

### Analysis of the results

In this set of experiments, the temperature of the cryostat is set to the steepest part of the superconducting transition (*T* ≈ 6.88 K), and three long-duration sweeps are made driving the plate to resonance from low to high frequency until it abruptly loses amplitude due to its nonlinear resonant response. This nonlinearity is well described by a simple Duffing equation with spring stiffening due to the mechanics of the doubly clamped plate. More general descriptions that include the electrostatic driving, roughness, or Casimir force^27^ are not required. An important feature in the plate response is the evidence in all three trials of a sudden clear deviation from the Duffing-nonlinear oscillator model in the amplitude and phase behavior occurring at ~811.2 kHz. This finding is due to the onset of the plate interacting with the oxide stops. Any additional nonlinearities in the plate mechanics due to deformation alone does not appear in this discontinuous manner but instead likely appears gradually. In addition, the amplitude is nearly fixed beyond this point, which is a further indication of contact. Upon contacting the pillars, the maximum peak displacement of the most deflected point on the plate centerline is estimated to be between 183 and 193 nm, resulting in a minimum Casimir cavity size of between 63 and 73 nm. Along the entire length of the superconducting sample and plate center, we estimate the average gap size at the closest approach to be between 85 and 141 nm. After the plate reaches its maximum amplitude at resonance (~815 kHz), the plate amplitude abruptly drops to zero, and the undeflected Casimir cavity size returns to ≈256 nm. This jump is where we might expect to see more clearly a corresponding change in the sample resistance if there were indeed a dependence of the superconducting transition temperature on the cavity size; however, no such statistically significant correlation is observed.

Another feature of the high-frequency Δ*T* measurements is seemingly random jumps of (300–400) µK. The cause of these instabilities is unknown; however, based on analyzing the three trials together, we do not believe they are related to the plate position. For example, in trial #1, we observe a large displacement at 820 kHz, which is well after the large plate oscillations have ceased. In trial #2, there is a large jump that is interestingly close to the minimum gap range, but a closer inspection of the data in Fig. 5 shows that it lags the plate contact by 4 or 5 data points (corresponding to 6–7.5 min of time difference), which is thus highly unlikely to be the effect we are looking for. Finally, in trial #3, Δ*T* appears to be tracking the plate amplitude, but then after the oscillations jump down, the signal does not follow. While undesirable, it is reasonable to assume that these intermittent jumps did not obscure the correlation between the abrupt change in the plate amplitude and the sample measurement. Rather, it is the underlying stochastic noise, indicated by the spread of the data of the individual areas shown in the green boxes in Fig. 4, which determines the experimental resolution.

To accurately quantify the uncertainty of the temperature measurement, we first consider this stochastic spread of the raw data as well as the uncertainty in the slope of the transition. For this value, we use the lower 95% confidence bound of the slope (11 kΩ/K) to conservatively claim a resolution. Using one standard deviation from each data set shown in the green areas of Fig. 4, averaging these with a weight prescribed by the number of data points in each set, and dividing by the lower estimate of the slope, we calculate one standard deviation per data point of 28 µK.

We look for the change in the critical temperature at the point of the abrupt change from a high amplitude vibration to the almost negligible vibration of the plate. At this jump, we quantify the change in the measured data by averaging 4 individual Δ*T* points immediately before and immediately after the jump and subtracting the averaged values. The average difference from the three experimental runs is (7 ± 12) µK. Thus, any effect of plate position on the transition temperature of the superconductor in our system is well below the one standard deviation statistical uncertainty of 12 µK.

### Interpretation of the experiment

In all three trials, no clear correlation between the plate amplitude or position and transition temperature was observed above our measurement resolution of 28 µK, and from the three trials combined, no change in *T*_c_ was observed to exceed the one standard deviation uncertainty of 12 µK. There are a few reasons that may explain this null result. No observation of this effect may be due to one or more of the following: (1) geometrical limitations, (2) superconductor limitations, and (3) theoretical uncertainties.

In the first case, the device geometry may not allow for a sufficiently small cavity to clearly observe a shift. The minimum cavity size that we are able to reach with the current configuration is at best on the order of 70 nm. Although this brings us into a theoretically interesting range, it may not be small enough to produce a measurable shift with the current materials. The uncertainty in the exact gap size exists due to the uncertain stress in the structure near 7 K, which is expanded upon in the “Methods” section. Using a basic scaling law shown in Eq. (8) (derived from calculations performed in ref. ^18^), we estimate the relative change in the Casimir-free energy between this minimum cavity size and the undeformed cavity size to be ≈400%. Further optimization of the timed oxide undercut and geometric design may allow for smaller cavities, which increase the magnitude of the Casimir-free energy compared to the condensation energy of the Pb film. Additionally, as discussed in the “Methods” section, the resonant mode shape analysis of the Au plate indicates that there is likely some degree of bowing of the plate along the length of the sample at the time of the closest approach, reducing the parallelism of the Au and Pb surfaces and thus the overall Casimir interaction.

Regarding the superconducting film itself, there are certain key material properties to consider when conducting this type of experiment. First, a low *T*_c_ value is generally desired because the condensation energy scales ∝ *T*_c_^2.6^^28^. As discussed, it is the ratio of the Casimir-free energy to the condensation energy that determines the magnitude of the shift in the critical field, so generally speaking, the lower *T*_c_ is, the better. In the case of the experiment presented here, Pb has a relatively high *T*_c_ value but was chosen for other experimental advantages. Future work may involve investigating lower *T*_c_ materials. Another important material property is the plasma frequency. Bimonte et al.^18^ showed that high plasma frequency materials can change the strength of the Casimir-free energy term by almost one order of magnitude. Many of the calculations in their work use are, with a plasma frequency of ~18 eV, which is higher than Pb, with a value of ~8 eV^29^. This reduction in the reflectivity at higher frequencies may result in a Casimir energy contribution to the free energy of condensation that is too small to observe.

Finally, there is the question of what we expect theoretically in the limit of zero applied magnetic field. The calculation methods used in this low field limit are not possible due to the condensation energy and the change in the free energy becoming comparable^30^. It is therefore not exactly clear what one might expect in terms of the magnitude of the change in the critical temperature as a result of a Casimir energy variation. Our experiment shows that for our geometry, materials, and in the range of temperatures we can resolve, there is no observed effect. Most certainly, the next steps in this project will involve extending the experiment to include magnetic characterization and extending the existing theory to the *H* = 0 case^31^.

## Conclusions

We have developed a unique nanomechanical transducer-based measurement technique and have undertaken a careful series of experiments to directly measure the shifts in the Casimir energy by placing a superconducting Pb film in a cavity and tuning the gap, looking for effects on the superconducting transition temperature of the film. Our chip-scale system can deposit and measure a superconducting thin film while simultaneously actuating a nearby plate, forming a tunable Casimir cavity. The in situ deposition process is achieved with two arrays of MEMS heaters that have been preloaded with a thick film of Pb and can be pulsed at low temperature to evaporate small amounts of material. The thin film is produced by using a shadow mask to define a precise pattern of the evaporated Pb (incident on the mask from two sides) that connects the four metallic measurement leads and creates a thin section of Pb directly underneath the movable Au plate. By driving a current with two sets of leads and measuring the voltage drop with the other two, we can measure the resistance of Pb. We monitor this resistance as the Au plate is driven to its mechanical resonance and back to zero amplitude.

Using finite element analysis, we are able to estimate the deformed mode shape at resonance, which places the minimum separation of Pb and Au between 63 and 73 nm. We estimate that at this gap size, the presence of the Au plate may be expected to produce the relative changes in the Casimir-free energy of the Pb film of 2× to 4×; however, a quantitative prediction for the corresponding change in *T*_c_ is a subject of ongoing theoretical work^31^ and is not available at present. The results presented here, at zero applied magnetic field, indicate that there are no observed Casimir-induced changes in *T*_c_ > 12 µK. By opening a novel experimental window regarding the relationship between the Casimir energy and superconductivity, we hope this work will stimulate further experimental and theoretical developments.

## Methods

### In situ film deposition and characterization

An experiment is performed in a closed-cycle cryostat specially designed for low mechanical vibrations. A typical experiment is performed as follows: (1) The source and target are mounted opposite one another and cooled to ≈3 K in the closed cycle system, (2) at low temperatures, Pb is evaporated onto the substrate, producing a smooth, continuous superconducting film, (3) the resistance of the Pb film is measured across its transition, and (4) the spacing of the Casimir cavity is modulated by applying an alternating voltage between the drive electrode and the suspended Au plate while the temperature of the system is held at the shoulder of the superconducting transition.

The resistance and transition of the Pb film are initially measured using a four-point configuration, shown schematically in Fig. 3 (inset). An excitation current, *I*_ex_, is applied at lead *I*+ (in series with an external 1 MΩ resistor) and travels through the central portion of the sample to *I*−, which is grounded through a 1 kΩ resistor. A voltage difference is then measured between the other two leads, *V*+ and *V*−. *I*_ex_ is a low-frequency AC current of ≈5 nA at 37.7 Hz. The differential voltage measurement is measured using a lock-in amplifier, and the resistance of the sample is then *V*_diff_/*I*_ex_. The temperature of the system is slowly swept across the transition (both down and up), which provides the slope of the transition, d*R*_s_/d*T*, as well as *T*_c_.

### Plate actuation and high-frequency measurement

The dynamic detection scheme involves measuring *V*_diff_ at the same frequency the plate is moving. First, we set the cryostat temperature to just below *T*_c_, where the resistance measurement is the most sensitive to the changes in temperature. Next, if the position of the plate does indeed influence the sample resistance due to the Casimir effect, then we expect a small modulation of *R*_s_ at the plate frequency. Subsequently, the voltage difference measured is as follows:

3 $$V_{{\mathrm{diff}}} = I_{{\mathrm{ex}}} \cdot \left( {R_{{\mathrm{s,0}}} + \Delta R\left( t \right)} \right)$$

where *R*_s,0_ is the nominal sample resistance and Δ*R*(*t*) is the small variation due to the plate. We can further breakdown Δ*R*:

4 $$\Delta {{R}} = \underbrace{\frac{{\partial R}}{{\partial T}}}_{{\rm{transition}}\,{\rm{slope}}} \cdot \underbrace{\frac{{\partial T}}{{\partial d}}}_{\rm{Casimir}} \cdot \underbrace{{\rm{d}}(t)}_{{\rm{plate}} \,{\rm{position}}}$$

The “Casimir” term is the theorized change in *T*_c_ of the Pb sample due to the changing position of the plate, d(*t*). This change is what we intend to detect. Thus, if we consider only the component of *V*_diff_ at the plate frequency, with amplitude |*V*_diff_|_plate_, then we can rearrange equation Δ*R* to obtain the amplitude of this expected effect:

5 $$\left| {\frac{{\partial T}}{{\partial d}}} \right| = \frac{{\left| {V_{{\mathrm{diff}}}} \right|_{{\mathrm{plate}}}}}{{\left| {I_{{\mathrm{ex}}}(t)} \right| \cdot \frac{{\partial R}}{{\partial T}} \cdot \left| {{\mathrm{d}}(t)} \right|}}$$

**Fig. 7:**
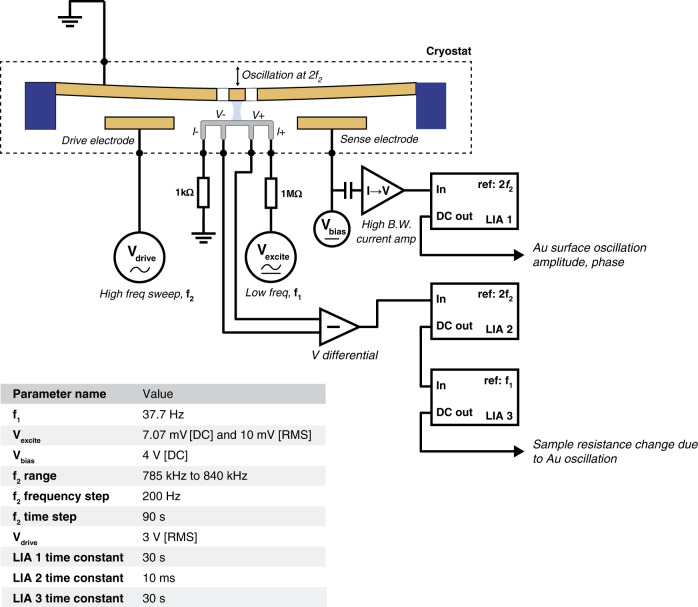
High-frequency detection circuit. The cryostat is held at a constant temperature just below *T*_c_ on the slope of the transition. The high-frequency *V*_drive_ signal is applied to the drive electrode and swept at frequency *f*_2_. The plate then feels an electrostatic force at frequency 2*f*_2_. The amplitude of the plate is monitored by measuring the AC current going through the sense electrode using a current-to-voltage amplifier and a lock-in referenced to 2*f*_2_ (LIA 1). The voltage drop across the Pb sample is also detected at 2*f*_2_. If there is any change in *T*_c_ due to the Casimir cavity size, then this is the frequency at which it would occur. Because the excitation current, *I*_ex_, is alternating at *f*_1_, the DC output of LIA 2 is fed into a third lock-in, LIA 3, which is referenced at *f*_1_

In this case, a more complex detection circuit is required. In Fig. 7, a high-frequency AC drive signal is used to actuate the plate at its resonance. The plate amplitude is detected at twice this drive frequency using LIA 1. Simultaneously, a current goes through the sample at a low frequency, while *V*_diff_ is being measured at the same frequency as the plate using LIA 2. The output of LIA 2 is then fed into LIA 3, which is locked into the low frequency of the excitation signal. The DC output of LIA 3 is then equal to the amplitude |*V*_diff_|_plate_. The inset table in Fig. 7 reports nominal values of each parameter presented in the schematic.

### Variation in the Casimir-free energy due to plate motion

It is possible to estimate the relative variation in the Casimir-free energy difference using the result from ref. ^18^

6 $$\Delta E_{{\mathrm{Cas}}} \propto \frac{1}{{1 + \left( {\frac{d}{{d_0}}} \right)^{1.15}}}$$

where *d* is the separation between the superconductor and metal and *d*_*0*_ is the nominal separation (≈256 nm in our experiment). It should be noted, however, that ref. ^18^ approximates the relation shown in Eq. (6) using a system, where *T*_c_ = 0.5 K, the superconductor thickness is 5 nm, and *d*_0_ = 8.3 nm, which is different than the system presented here. Considering the relative change in Δ*E*_Cas_ to be δ*E*_Cas_ = Δ*E*_Cas_(*d*)/Δ*E*_Cas_(*d*_0_) with d(*t*) changing sinusoidally at *ω* = 2*πf*_2_ with amplitude *A*, we find:

7 $$\delta E_{{\mathrm{Cas}}}(t,A) = \frac{2}{{1 + \left( {\frac{{d_0 + A\sin (\omega t)}}{{d_0}}} \right)^{1.15}}}$$

To a first approximation, we assume that δ*E*_Cas_(*t*) varies *T*_c_(*t*) linearly. Because the phase-sensitive detection used in the experiment only measures the time-averaged component of the signal at ω, we can approximate Eq. (7) by considering only the time-averaged magnitude of the first harmonic term as a function of *A*. Numerically solving for the magnitude of the first Fourier coefficient of δ*E*_Cas_(*t*, *A*) over the range *A* = 0–200 nm and using *d*_0_ = 256 nm, we find that it can be well approximated by a third-order polynomial:

8 $$\left| {\delta E_{{\mathrm{Cas}}}} \right| = c_1A + c_2A^3$$

where *c*_1_ = 0.0225 nm^−1^ and *c*_2_ = 5.905 × 10^−9^ nm^−3^. Using Eq. (8), we can then estimate the relative change in the Casimir-free energy for a given plate amplitude. This estimate considers two parallel areas, separated by *d*. In reality, the interaction of the Au plate and Pb sample is not parallel, and the geometry is defined by the deformed shape of the plate. An exact analytical calculation of the Casimir interactions between general, nonparallel shapes is an open problem, which we do not address in this analysis. However, a study of how the plate might be deforming at its resonance is discussed in the following section.

### Estimating the plate deflection and mode shape using finite element analysis

Determining the exact amplitude of the plate and therefore the Casimir gap size is challenging due to a significant change in the dynamics that occurs between room temperature and the cryogenic temperature. This behavior is demonstrated by a large change in the resonant frequency, from ≈700 kHz to ≈1.8 MHz between 300 and 3 K, respectively, indicating the substantial tensile stress that occurs in the top Au layer due to thermal contraction of the Au relative to the substrate. By examining the behavior of the signal at the sense electrode, it is possible to determine when contact with the oxide pillars occurs; however, determining the distance between the Pb sample and the center of the plate (parallel to the Pb sample) requires knowledge of the deformed shape of the Au plate at its resonance.

Qualitatively, a Duffing response nonlinearity arises due to the dynamic increase in the tension of the vibrating Au plate, which slightly increases the mean resonance frequency with amplitude. However, because this change in the dynamic stress over an oscillation cycle is much smaller than the static stress, the mode shape is not much different than that of a linear, prestressed structure. Using a commercial finite element software package to calculate mechanical eigenmodes, three different initial stress cases are considered to place bounds on the distance at which the center of the plate deflects at resonance. The first case considers zero initial stress, and the second and third cases include two different values of a uniaxially applied initial stress (0.1 and 0.26 GPa, respectively).

**Fig. 8:**
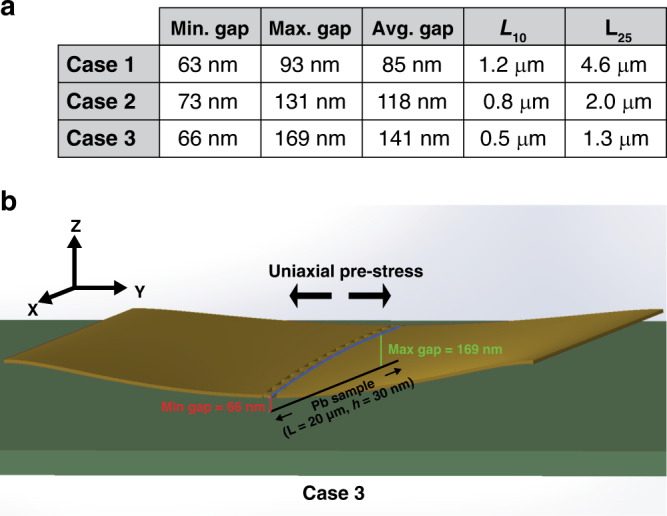
Finite element analysis for determining the mode shape and cavity size. **a** Results from the finite element analysis for gap sizes achieved along the length of the Pb sample (20 µm in length) upon contact between the deformed Au plate and the oxide pillars. With no actuation, the gap size is assumed to be 256 nm (sample thickness subtracted from oxide thickness). **b** 3D representation of the gap size estimation using finite element results for case 3 (high uniaxial stress). In this case, the profile of the plate along the sample (blue line) is asymmetric and reaches much closer to the sample on one side compared to the other. This reduces the average gap size as well as L_10_ and L_25_

After a mode shape is obtained, it is scaled in amplitude (*Z*) until any part of the surface comes into contact with any of the oxide pillars, and then the profile parallel to the sample is extracted. In Fig. 8a, the values of maximum separation, minimum separation, and average separation are reported as well as the two lengths: L_10_ and L_25_, which are the lengths of the sample that are within 10% and 25%, respectively, of the minimum gap size.

*Case 1*: Although there is clearly evidence of significant initial stress in the movable Au layer, it is necessary to estimate the upper bound of the plate deflection by considering the zero stress case. Solving for the first eigenfrequency of the device geometry returns a value of 470 kHz, which is, as expected, well below the measured 1.8 MHz.

*Case 2*: Clearly, for the simulation to match the measured resonant frequency, a prestress must be applied. The reason for the two different stress cases 2 and 3 is that in the high-stress limit, finite element analysis suggests that the fundamental mode and the second mode become near degenerate and mix due to the slight layout asymmetry in the center cut of the moving plate. This resulting high-stress mode shape is essentially only one side of the cavity moving up and down, while the other side moves with a much smaller amplitude. Case 2 considers an intermediate stress case before this degeneracy is reached, and the shape of the fundamental mode still resembles that of the zero stress case. In this simulation, the uniaxial prestress is equal to 0.1 GPa, and the resulting resonant frequency is 1.12 MHz.

*Case 3*: In this simulation, the uniaxial prestress is increased until we reach a resonant frequency that matches the experiment (≈1.8 MHz). This stress is equal to 0.26 GPa and is generally consistent with the estimates from the differential thermal expansion. However, in this high-stress condition, the mode shape is very asymmetric due to the mixing of the fundamental and the second modes. As a result, only one edge of the Au displaces enough to become close to the sample (Fig. 8b).

A full understanding of the exact shape of the Au plate at its resonance is not possible; however, by using the measured current amplitude from the experiment in conjunction with post-experimental finite element analysis, we can infer that the minimum separation of the Pb sample and the Au plate is likely between 63 and 73 nm and that the average separation across the length of the sample is likely between 85 and 141 nm. Using the estimated scaling dependence shown in Eq. (8), these deflections would produce relative changes in the Casimir-free energy in the Pb sample between 415% and 439% (when considering only the area near the minimum gap) and between 260% and 388% when considering the average gap over the length of the sample.

## Supplementary information

Supplementary Information

## References

[1] Casimir HB (1948). On the attraction between two perfectly conducting plates. Proc. K. Ned. Akad. Wet..

[2] Lamoreaux SK (1997). Demonstration of the Casimir force in the 0. 6 to 6 μm range. Phys. Rev. Lett..

[3] Mohideen U, Roy A (1998). Precision measurement of the Casimir force from 0.1 to 0.9 μm. Phys. Rev. Lett..

[4] Chan HB, Aksyuk VA, Kleiman RN, Bishop DJ, Capasso F (2001). Quantum mechanical actuation of microelectromechanical systems by the Casimir force. Science.

[5] Chan HB, Aksyuk VA, Kleiman RN, Bishop DJ, Capasso F (2001). Nonlinear micromechanical Casimir oscillator. Phys. Rev. Lett..

[6] Tang L (2017). Measurement of non-monotonic Casimir forces between silicon nanostructures. Nat. Photonics.

[7] Antezza M (2020). Giant Casimir torque between rotated gratings and the θ=0 anomaly. Phys. Rev. Lett..

[8] Stange A, Imboden M, Javor J, Barrett L, Bishop DJ (2019). Building a Casimir metrology platform with a commercial MEMS sensor. Microsyst. Nanoeng..

[9] Jourdan G, Lambrecht A, Comin F, Chevrier J (2009). Quantitative non-contact dynamic Casimir force measurements. EPL.

[10] Imboden M, Morrison J, Campbell D, Bishop D (2014). Design of a Casimir-driven parametric amplifier. J. Appl. Phys..

[11] Munday J, Capasso F, Parsegian V (2009). Measured long-range repulsive Casimir–Lifshitz forces. Nature.

[12] Levin M, McCauley AP, Rodriguez AW, Reid MTH, Johnson SG (2010). Casimir repulsion between metallic objects in vacuum. Phys. Rev. Lett..

[13] Intravaia F (2013). Strong Casimir force reduction through metallic surface nanostructuring. Nat. Commun..

[14] Bimonte G, Lopez D, Decca R (2016). Isoelectronic determination of the thermal Casimir force. Phys. Rev..

[15] Glover RE, Tinkham M (1957). Conductivity of superconducting films for photon energies between 0.3 and 40 kT_c_. Phys. Rev..

[16] Norte RA, Forsch M, Wallucks A, Marinković I, Gröblacher S (2018). Platform for measurements of the Casimir force between two superconductors. Phys. Rev. Lett..

[17] Bimonte G, Calloni E, Esposito G, Milano L, Rosa L (2005). Towards measuring variations of Casimir energy by a superconducting cavity. Phys. Rev. Lett..

[18] Bimonte G, Calloni E, Esposito G, Rosa L (2005). Variations of Casimir energy from a superconducting transition. Nucl. Phys. B.

[19] Neugebauer CA, Webb MB (1962). Electrical conduction mechanism in ultrathin, evaporated metal films. J. Appl. Phys..

[20] Ekinci KL, Valles JM (1999). Morphology of quench condensed Pb Films near the insulator to metal transition. Phys. Rev. Lett..

[21] Imboden M, Han H, Stark T, Bishop D (2017). Cryogenic fab on a chip sticks the landing. ACS Nano.

[22] Imboden M (2013). Atomic calligraphy: the direct writing of nanoscale structures using MEMS. Nano Lett..

[23] Imboden M (2014). Building a fab on a chip. Nanoscale.

[24] Imboden M, Bishop D (2014). Top-down nanomanufacturing. Phys. Today.

[25] Han H (2015). Programmable solid state atom sources for nanofabrication. Nanoscale.

[26] Franck JP, Martin DL (1961). The superconducting transition temperature of lead. Can. J. Phys..

[27] Broer W, Waalkens H, Svetovoy VB, Knoester J, Palasantzas G (2015). Nonlinear actuation dynamics of driven casimir oscillators with rough surfaces. Phys. Rev. Appl..

[28] Lewis HW (1956). Superconductivity and electronic specific heat. Phys. Rev..

[29] Ordal MA, Bell RJ, Alexander RW, Long LL, Querry MR (1985). Optical properties of fourteen metals in the infrared and far infrared: Al, Co, Cu, Au, Fe, Pb, Mo, Ni, Pd, Pt, Ag, Ti, V, and W. Appl. Opt..

[30] Bimonte G (2008). Low noise cryogenic system for the measurement of the Casimir energy in rigid cavities. J. Phys. A.

[31] Som, A., Aksyuk, V., Barrett, L. K., Imboden, M., Lally, R. K., Pérez-Morelo, D., Stange, A., Bishop, D. J. & Campbell, D. K. On-going work (2020). 10.1038/s41378-020-00221-2 PMC7767790 33414928

