# Supplementary material for: A system for probing Casimir energy corrections to the condensation energy
Source: Microsyst Nanoeng. 2020 Dec 28;6:115. doi: 10.1038/s41378-020-00221-2 (PMC7767790; doi:10.1038/s41378-020-00221-2)
Supplement: Supplementary file 1 — Supplementary Information [file 41378_2020_221_MOESM1_ESM.docx]

**Supplementary information**

**Target die design and fabrication**

**Figure S1**: Fabrication steps of the nano-mechanical target die.


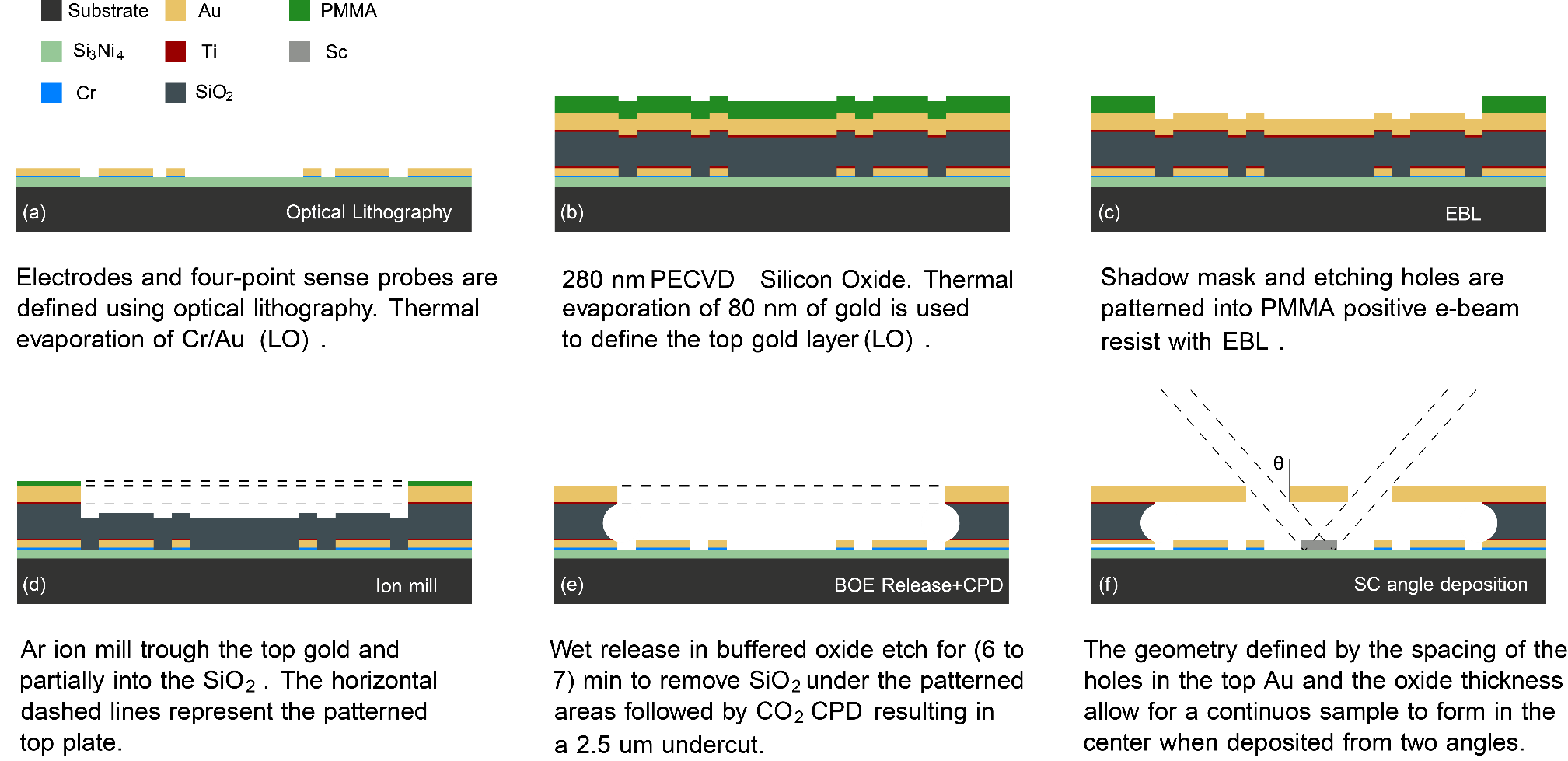


The target die contains the substrate with pre-positioned electrical leads along with a movable Au plate suspended above. This Au plate serves as both the *in situ* evaporation mask as well as tunable half of the Casimir cavity. Detailed fabrication steps can be found below as well as in ref. [1].

Figures S1a-e illustrate the process flow that has been used in the nanofabrication of the device, which leverages non-contact optical and electron-beam lithography steps to define the devices features. Devices are fabricated monolithically from bare Silicon wafer. In the first step, we deposit nominally 100 nm of Silicon Nitride using low pressure chemical deposition (LPCVD) as electrical isolation between the metallic layers and the substrate. Next, the bottom metal layer consisting of nominally 10 nm Cr, 40 nm Au, and 2 nm Ti is defined using optical lithography followed by electron beam evaporation and lift-off (LO). This layer defines two electrodes for electrostatic actuation and four-probe connections for *in situ* monitoring of the superconductor electrical resistance. Electrical leads wire each feature to bonding pads in a way that each element can be accessed independently.

Next, plasma-enhanced chemical vapor deposition (PECVD) at 180 °C is used to deposit SiO_2_. The oxide was used both as a sacrificial layer and structural layer to define the oxide pillars.

Following the oxide deposition, a second metallic layer consisting of nominally 2 nm Ti and 80 nm Au is formed in the same way as the first layer. Au windows positioned above electrical pads are obtained from by this lift-off step, which are later used as a shadow mask to prevent shorting during the superconductor evaporation.

The top Au is then lithographically patterned for the second time using poly (methyl methacrylate) (PMMA) positive electron beam resist and electron beam lithography (EBL). The pattern is then transferred through to the Au layer with anisotropic argon ion milling, with the wafer rotating, the stage cooled to 10 °C and the incidence angle 10° off normal [1].

Finally, 1.25 mm x 1.25 mm chips, each containing one centered device, are singulated using a dicing saw. Movable structures in the top Au layer were released by wet etching in a buffered oxide etch (BOE) 6:1 solution for (6 to 7) min followed by sequential rinsing in water and isopropyl alcohol (IPA) baths. The resultant Silicon Oxide lateral undercut distance is about 2.5 µm. Finally, to prevent the suspended top gold from sticking to the bottom gold, the device was dried in a CO_2_ critical point dryer (CPD). Note that after BOE step both Ti layers are wet etched from bottom and top of the corresponding Au layers.

In figure S1f, the final target structure and corresponding geometry is presented. The EBL pattern etched into the top Au plate (shown in black) is designed to form a continuous thin film of Pb in a ‘H’ shaped pattern (see figure 2 in the main text) for an angled deposition at θ = 35°. This corresponds to a center-to-center spacing of the micro-source of 1.1 mm. The ‘H’ pattern will connect the four-point measurement leads as well as create one continuous line of Pb down the center of the cavity, beneath a continuous section of suspended Au.

**Micro-source fabrication, preparation, and configuration**

**
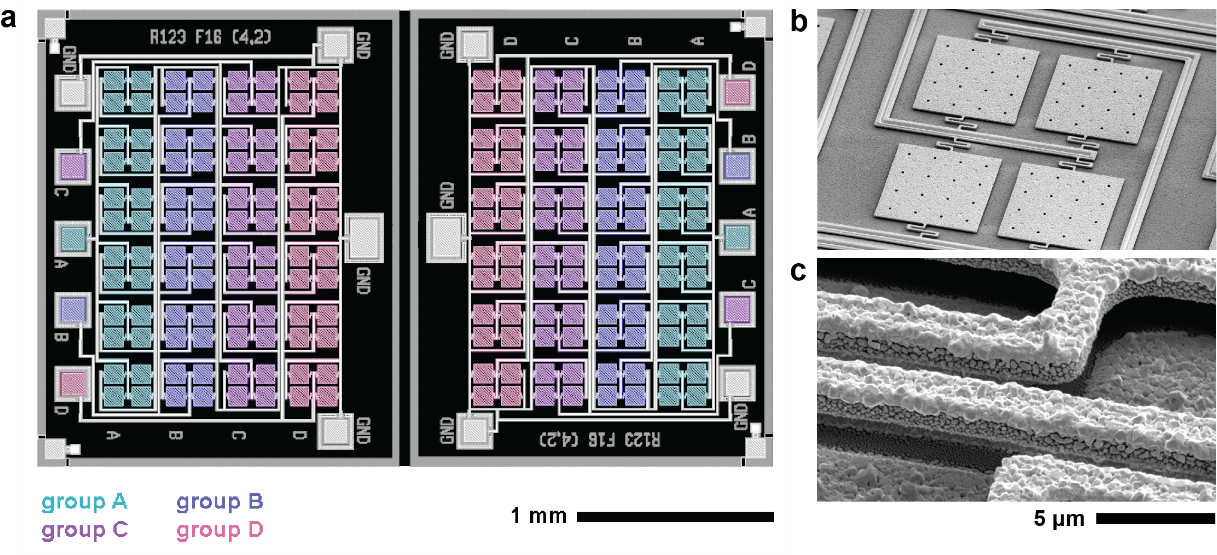
**

**Figure S2:** Micro-sources for quenched-condensed Pb deposition. **a.** Images of MEMS design files arranged to depict the actual micro-source configuration in the experiment. Two sets of dies, a left and a right, each contain 4 individually addressable groups of heaters, labelled A, B, C, D and color coded. By pulsing current through each group to ground (white bonding pads), we can sequentially deposit Pb from large angles towards small angles. **b.** SEM image of one set of 4 heaters. Current runs from the center of the quadrant in parallel through each heater to ground through serpentine connections. **c.** Zoomed in SEM image of the serpentine connection with Pb already loaded. The light material laying on top is Pb (about 750 nm thick) and the suspended poly-silicon structure can be seen beneath it.

The evaporation of the Pb is done from two different dies, a left and a right. By adjusting the center-to-center spacing between the left and right die, one can tune the angle of evaporation through the mask (see figure 2). The mask geometry used on the target die is designed for a center to center spacing of ≈ 1.1 mm. Each source die is 2.5mm × 2mm and consists of four individually addressable groups, each containing 24 micro-source heater units. Current is run in parallel through each group of 24 heaters individually. Figure S2a shows a colorized mask design file depicting the layout of the two micro-source dies and each of the 8 groups. Fabrication of these micro-structures is detailed in ref. [2].

Each micro-source unit consists of a freely suspended square MEMS plate, 100x100 µm^2^ in area and 1.5 µm thick, connected with serpentine electrical leads (see figure S2b). The plates are only weakly thermally connected to the substrate they sit on and small amounts of power, typically using pulse width modulation (PWM), can be used to evaporate a small number of atoms per pulse. This can be done at low temperatures, with good control and minimal heating of the substrate upon which they land.

The actual center-to-center spacing of the micro-source dies is around 1.9 mm and the arrays are spaced over a distance of 2 mm which results in an angular deposition range of 24° to 58°. The flux reaching the target at around θ = 35° is what contributes to the Pb side of the cavity, while the flux at smaller and larger angles contributes to making a good connection between the Pb film and the four Au measurement leads. Prior to loading into the cryostat, 500 nm to 1000 nm of Pb is evaporated onto the micro-source dies by heating a resistive thermal crucible inside a vacuum chamber.

The technique used to evaporate Pb from the micro-sources involves applying very small, very short pulses of power to the heaters in order to sublime very small volumes of material from the evaporated Pb. In this regime, very low atomic fluxes can be achieved, and high quality, thin films can be deposited on the target. Voltage pulses are applied to each group of sources sequentially, starting from the outside (groups A) and moving inwards towards groups D (see figure S2a).

**References for supplementary information:**

1. Dennis, B., Haftel, M., Czaplewshi, D., Lopez, D., Blumberg, G. and Aksyuk, V. “Compact Nanomechanical Plasmonic Phase Modulators”. Nature Photonics **9**, 267-273 (2015).
2. Han, H., Imboden, M., Stark, T., Del Corro, P. G., Pardo, F., Bolle, C. A., Lally, R. W., and Bishop, D. J. “Programmable solid state atom sources for nanofabrication.” Nanoscale, **7** (24), 10735-10744, (2015).
